# Supplementary material for: Electron microscopy snapshots of single particles from single cells
Source: J Biol Chem. 2018 Dec 12;294(5):1602–8. doi: 10.1074/jbc.RA118.006686 (PMC6364765; doi:10.1074/jbc.RA118.006686)
Supplement: Supporting Information [file supp_294_5_1602__index.html]

Electron microscopy snapshots of single particles from single cells — Single-cell structural biology — Electron microscopy snapshots of single particles from single cells — Single-cell structural biology — Supporting Information 

# Electron microscopy snapshots of single particles from single cells

## Supporting Information

- Supporting Movie 1 - Video showing the process of embryo lysis and transfer of the lysate to a reference EM grid.
- Supporting Information (to be published online) - Supporting Information
