# Supplementary material for: Electron microscopy snapshots of single particles from single cells
Source: J Biol Chem. 2018 Dec 12;294(5):1602–8. doi: 10.1074/jbc.RA118.006686 (PMC6364765; doi:10.1074/jbc.RA118.006686)
Supplement: Supporting Information [file supp_RA118.006686_141920_1_supp_249380_pjkzwn.pdf]

Electron microscopy snapshots of single particles from single cells

Xiunan Yi[1-3*], Eric J. Verbeke[1-3*], Yiran Chang[1-3*], Daniel J. Dickinson[1–3†], David W. Taylor[1–4†]

From the [1]Department of Molecular Biosciences, [2]Center for Systems and Synthetic Biology
[3]Institute for Cellular and Molecular Biology, University of Texas at Austin, Austin, TX 78712;
[4]LIVESTRONG Cancer Institute, Dell Medical School, Austin, TX 78712

Running title: *Single-cell structural biology*

*These authors contributed equally to this work.

†To whom correspondence should be addressed: David W. Taylor: Department of Molecular Biosciences, University of Texas at Austin, Austin, TX 78712; dtaylor@utexas.edu; Tel. (512) 471-9156; and Daniel J. Dickinson: Department of Molecular Biosciences, University of Texas at Austin, Austin, TX 78712; daniel.dickinson@austin.utexas.edu; Tel. (512) 232-2916.

SUPPORTING INFORMATION

Figure S1: Lysate transfer control experiments and small particle classes.

Figure S2: RNA-seq data of *C. elegans* embryos.

Figure S3: Classification of ribosomes from single cells.

Figure S4: Reconstruction of an 80S ribosome and Fourier shell correlation curves.

Supporting movie 1: Video showing the process of embryo lysis and transfer of the lysate to a reference EM grid.

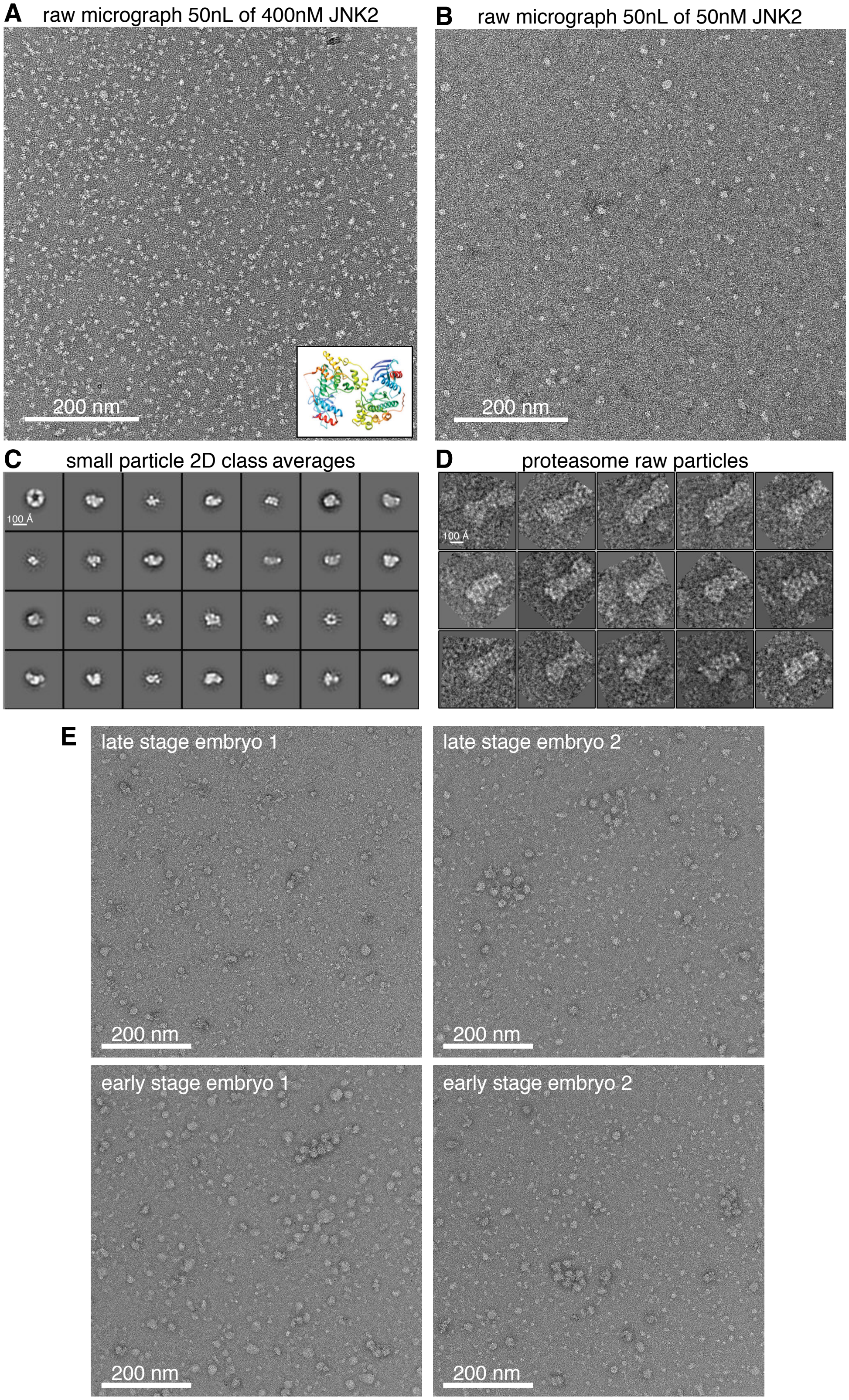

Figure S1: Lysate transfer control experiments and small particle classes

(A) Micrograph of 50nL of 400nM JNK2 (a kinase protein with known structure). To demonstrate that we can visualize particles using our method, we first flowed 400nM JNK2 solution through the PDMS channel

and then ~50nL of the solution was transferred to the reference grid using a glass needle. Inset, a view of the JNK x-ray crystal structure (PDB 3E7O) (33).
(B) Micrograph of 50nL of 50nM JNK2.
(C) Reference-free 2D class averages of small particles picked from two early-staged embryo datasets containing ~126,000 particles. Classes show unique structural features such as a pentameric ring in the top left corner.
(D) Individual raw particles of the 26S proteasome show distinct features directly from micrographs. Particles were visualized using the ‘Display’ and ‘Show particles in selected class’ graphical user interface within RELION. Particles have been rotated and translated based on 2D classification in RELION. Box size is 576 Å x 576 Å.
(E) Representative micrographs from multiple individual single cell experiments shows similar dispersion and size range of single particles.

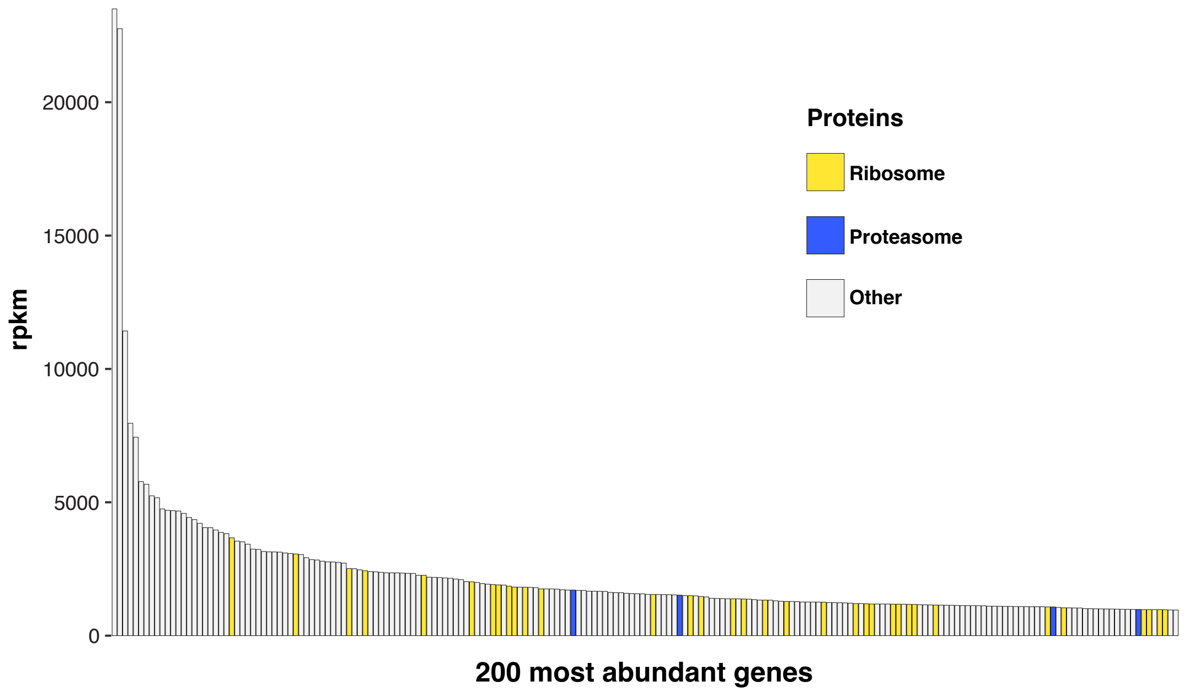

Figure S2: RNA-seq data of *C. elegans* embryos
The 200 most abundant genes in *C. elegans* zygotes (determined by RNAseq (16, 17)) sorted in order of decreasing abundance.

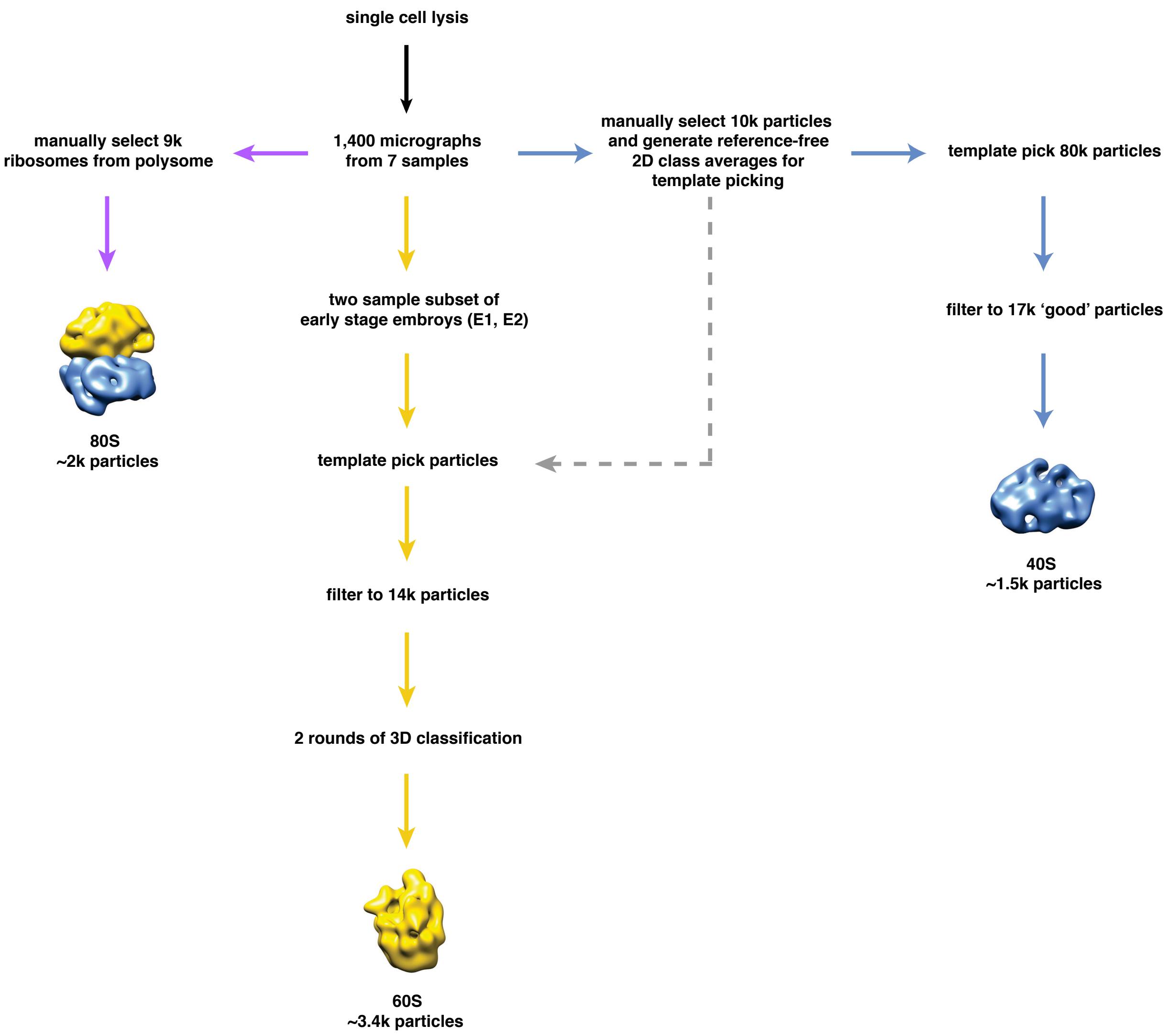

Figure S3: Classification of ribosomes from single cells
Workflow for classification of particles from single cells into 40S, 60S, and 80S ribosomes structures.

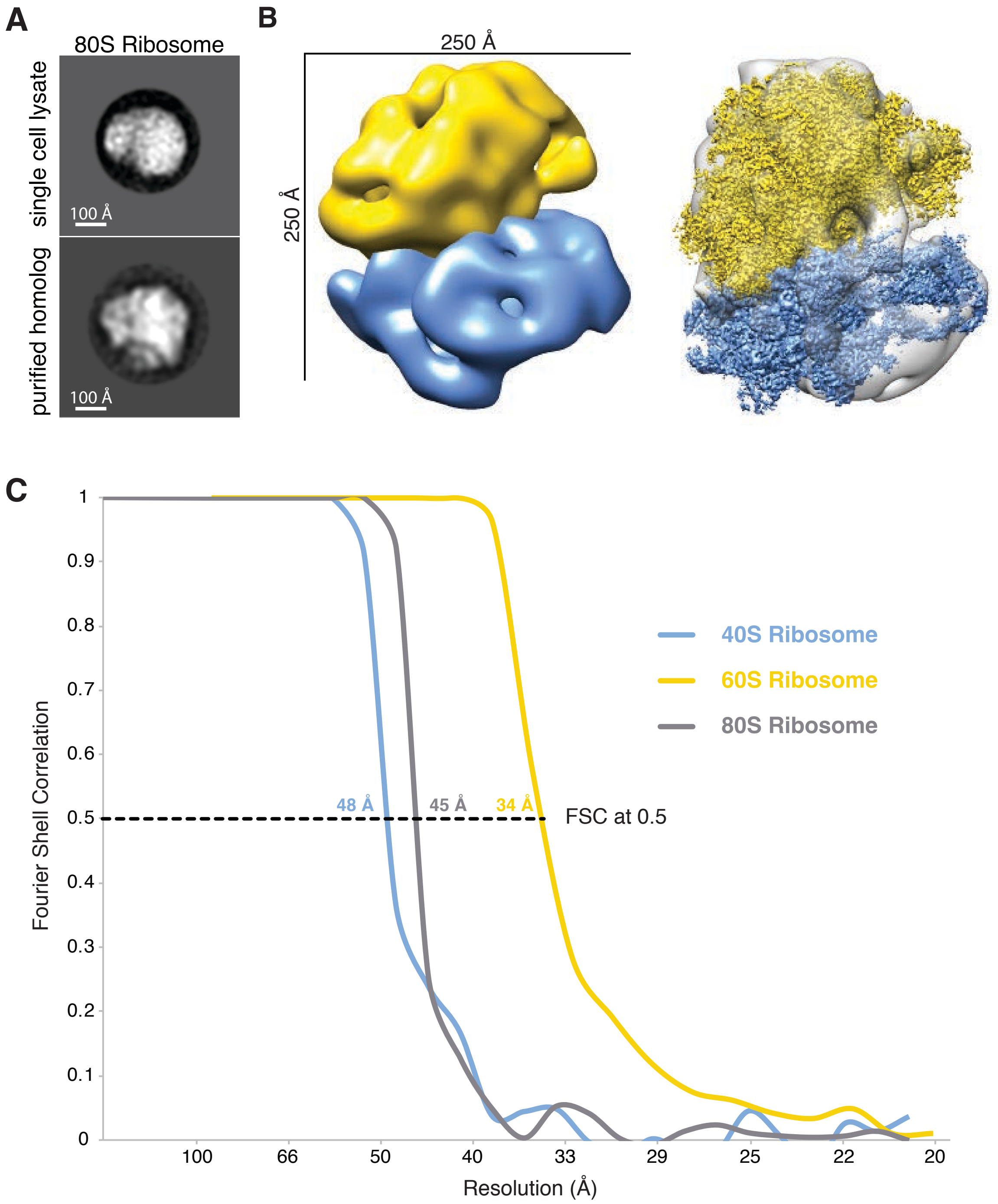

Figure S4: Reconstruction of an 80S ribosome and Fourier shell correlations

(A) Reference-free 2D class average from ribosomes in polysomes aligned to a purified homolog from *S. cerevisiae*.

(B) Left: Our 80S ribosome hybrid model. Right: Our 80S ribosome model reconstructed from ribosomes in polysomes with high-resolution 60S (EMDB-2811) (25) and 40S (EMDB-4214) (26) shown in yellow and blue, respectively.

(C) Fourier shell correlations of our 40S, 60S and 80S ribosome models. Nominal resolution values are reported at a correlation score of 0.5.
